# Supplementary figures and images for: Biologically-constrained spiking neural network for neuromodulation in locomotor recovery after spinal cord injury
Source: PLoS Comput Biol. 2026 Jan 6;22(1):e1013866. doi: 10.1371/journal.pcbi.1013866 (PMC12799191; doi:10.1371/journal.pcbi.1013866)

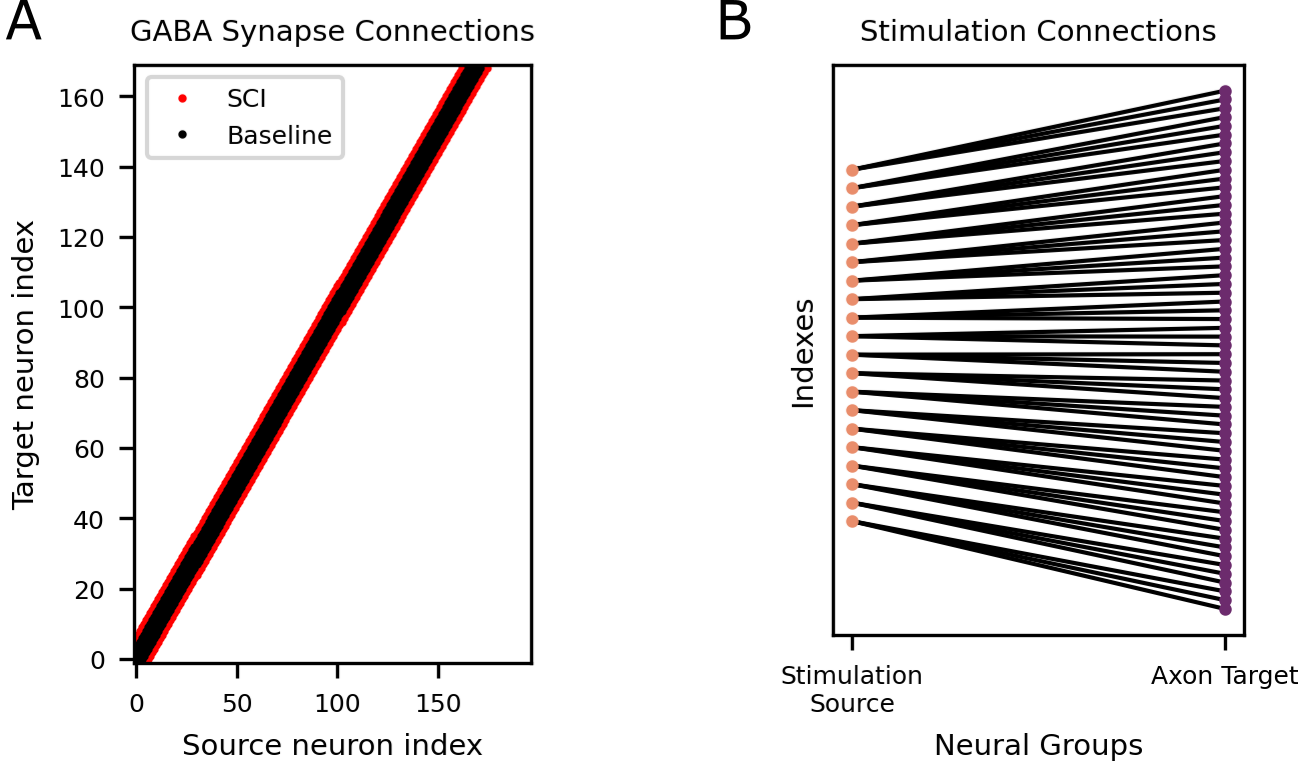

Supplement: S1 Fig — (TIFF) [file pcbi.1013866.s001.tiff]

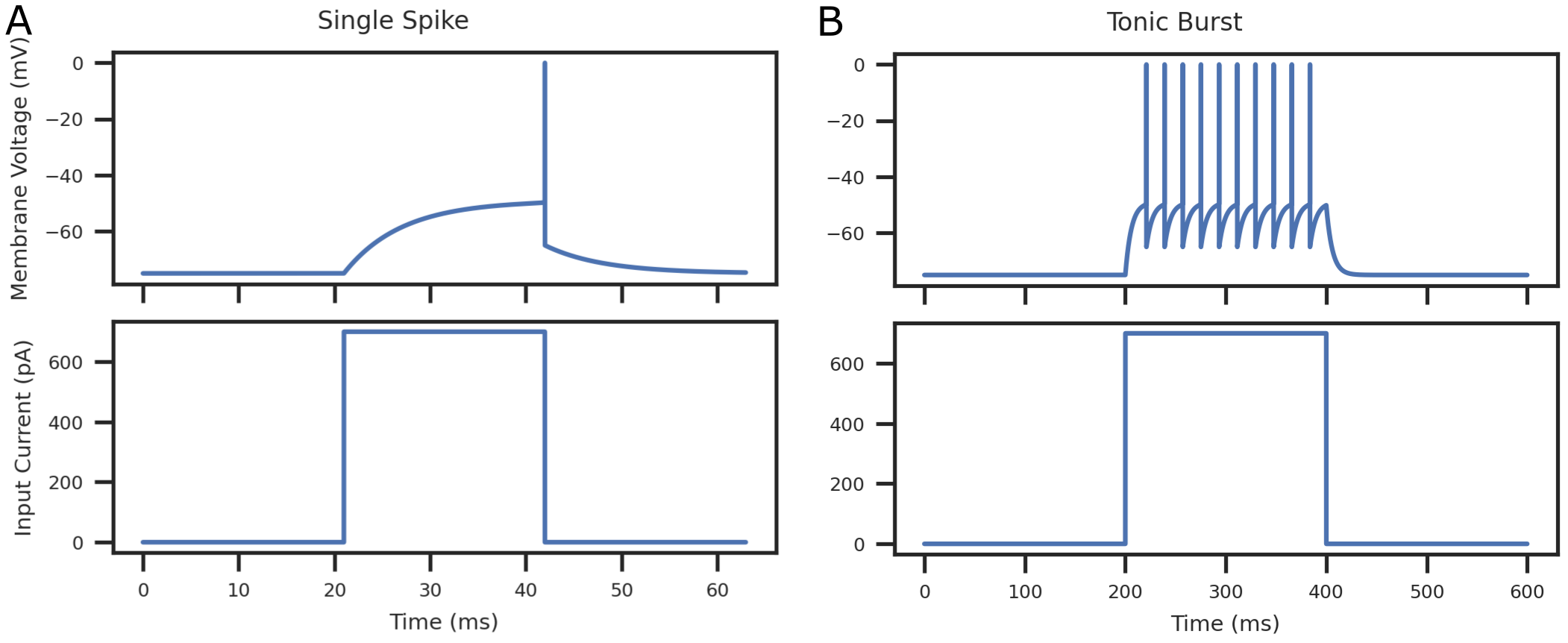

Supplement: S2 Fig — (A) and tonic burst (B) response after receiving a 20 ms and 200 ms stimulation pulse at 670 pA, respectively. (TIFF) [file pcbi.1013866.s005.tiff]

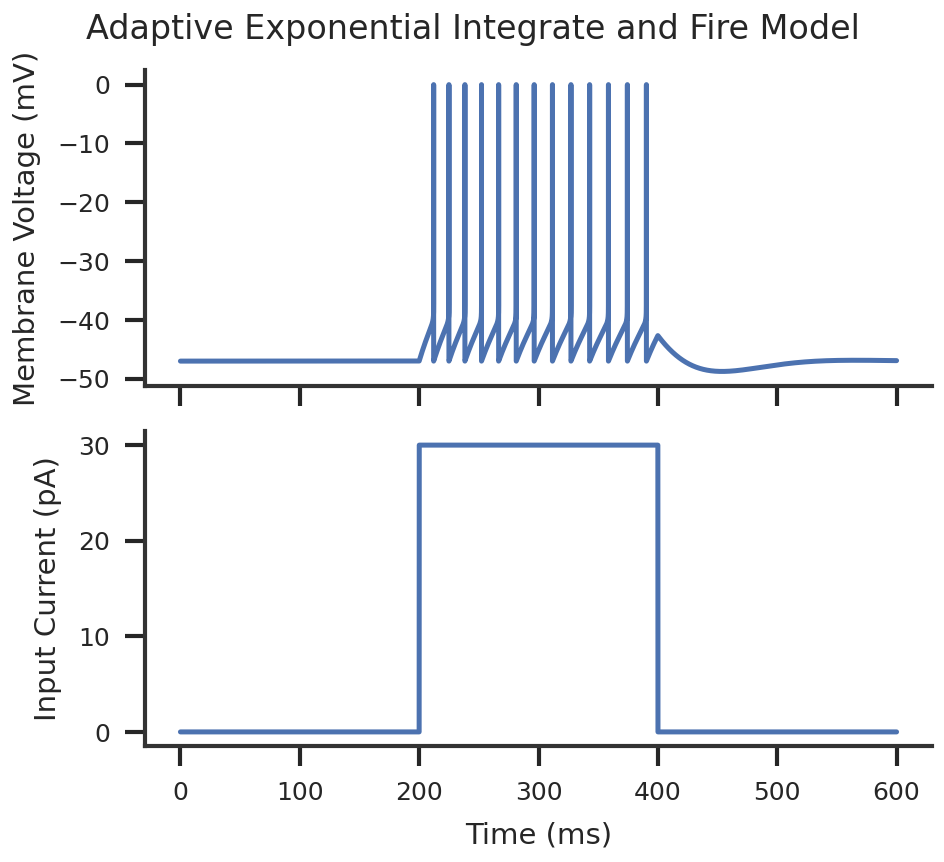

Supplement: S3 Fig — (TIFF) [file pcbi.1013866.s008.tiff]

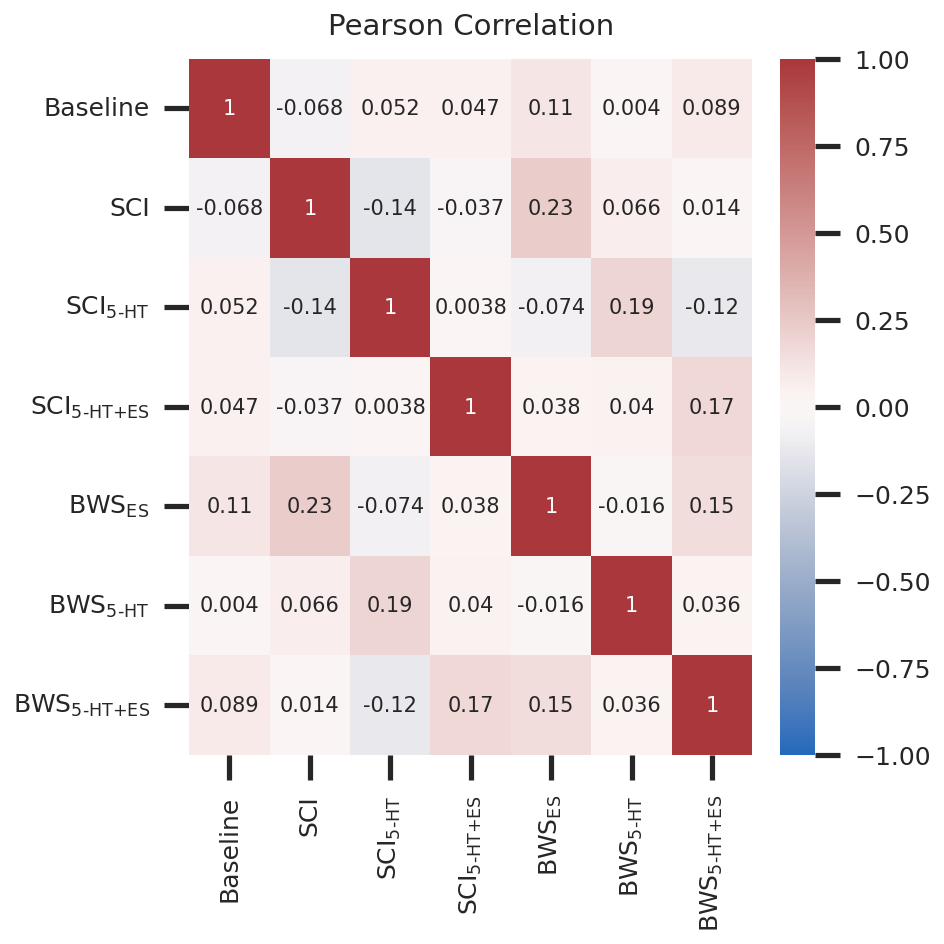

Supplement: S4 Fig — Steps were concatenated to a single array. All correlations were significant (p<0.0001). (TIFF) [file pcbi.1013866.s009.tiff]

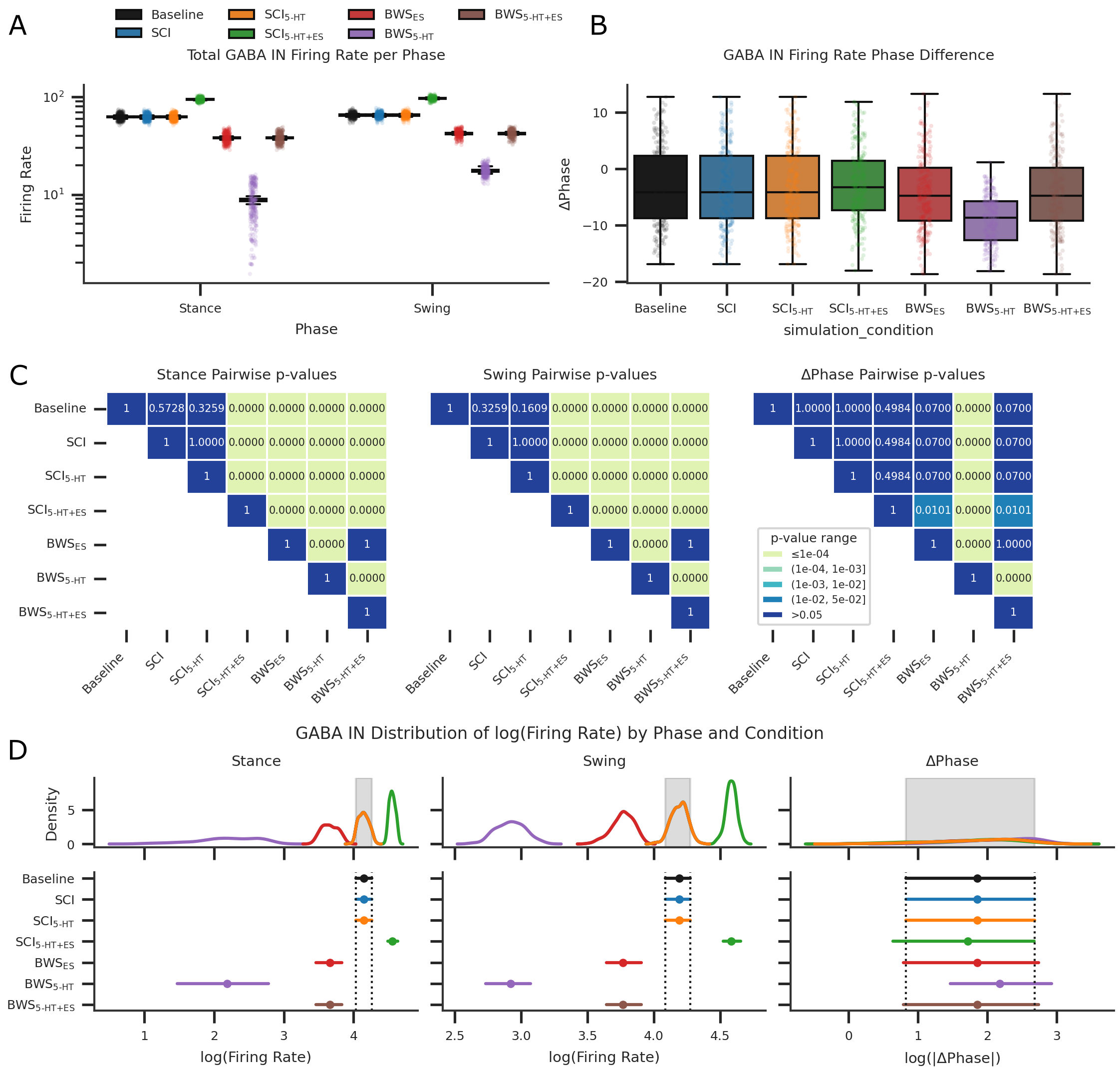

Supplement: S5 Fig — (TIFF) [file pcbi.1013866.s011.tiff]

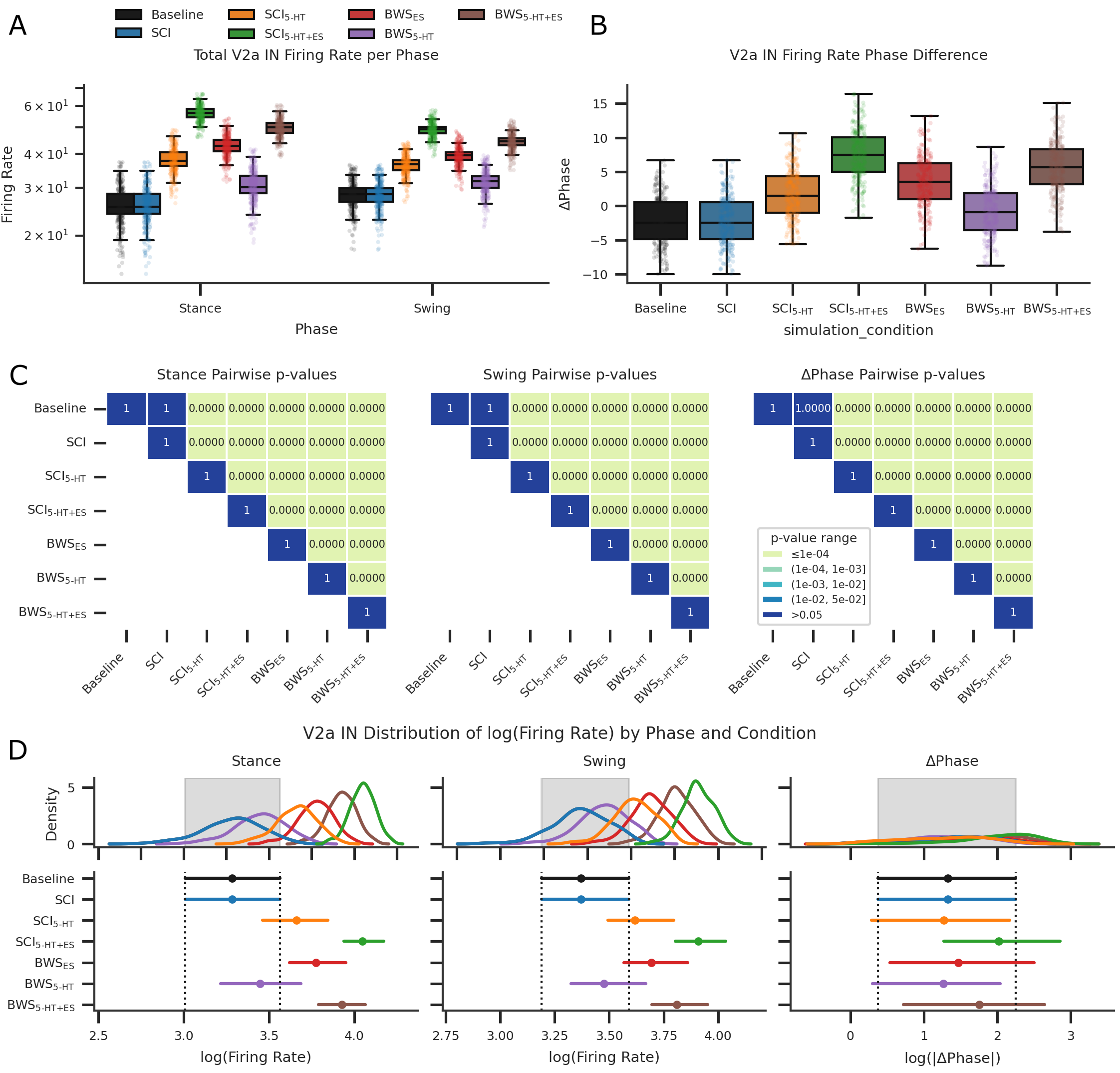

Supplement: S6 Fig — (TIFF) [file pcbi.1013866.s013.tiff]
