# Supplementary material for: Biologically-constrained spiking neural network for neuromodulation in locomotor recovery after spinal cord injury
Source: PLoS Comput Biol. 2026 Jan 6;22(1):e1013866. doi: 10.1371/journal.pcbi.1013866 (PMC12799191; doi:10.1371/journal.pcbi.1013866)
Supplement: S1 Algorithm — (PDF) [file pcbi.1013866.s002.pdf]

S1 Algorithm Leaky Integrate-and-Fire (LIF) neuron model used to simulate membrane potential dynamics. The membrane potential V evolves according to an exponential decay toward the reverse potential E_L, driven by input current $\mathrm{I}(t)$. A spike is emitted when $V \geq V_{th}$, after which V is reset to V_{reset}

Require: Membrane time constant τ_{mem}, reverse potential E_L, threshold V_{th}, reset potential V_{reset}, input current $I(t)$, time step Δt, total simulation time T

Ensure: Spike times $\mathcal{S}$

Initialize membrane potential $V \leftarrow V_{rest}$
Initialize spike train $\mathcal{S} \leftarrow \emptyset$
for $t = 0$ to T with step Δt **do**
    if $t_{ref} > 0$ **then**
        $t_{ref} \leftarrow t_{ref} - \Delta t$
        $V \leftarrow V_{reset}$ ▷ Hold membrane at reset during refractory period
    else
        Compute membrane potential derivative as per equations (3), (5) and (6)
        Update membrane potential:

$$V \leftarrow V + \frac{dV}{dt}\Delta t$$

        if $V \geq V_{th}$ **then**
            Record spike time $\mathcal{S} \leftarrow \mathcal{S} \cup \{t\}$
            Reset potential: $V \leftarrow V_{reset}$
            Enter refractory state: $t_{ref} \leftarrow \tau_{ref}$
        end if
    end if
end for
return $\mathcal{S}$
