# Supplementary material for: Biologically-constrained spiking neural network for neuromodulation in locomotor recovery after spinal cord injury
Source: PLoS Comput Biol. 2026 Jan 6;22(1):e1013866. doi: 10.1371/journal.pcbi.1013866 (PMC12799191; doi:10.1371/journal.pcbi.1013866)
Supplement: S1 Table — All correlations were significant (p < 0.05). (PDF) [file pcbi.1013866.s003.pdf]

S1 Table. TA and GM afferent axon tuning performance measured by Pearson correlation coefficient (CC) and mean absolute error (MAE). All correlations were significant ($p < 0.05$).

Afferent	CC	MAE (Hz)
TA Ia	0.99	3.07
TA II	0.99	2.38
GM Ia	1.00	3.13
GM II	0.99	2.12
