# Supplementary material for: Biologically-constrained spiking neural network for neuromodulation in locomotor recovery after spinal cord injury
Source: PLoS Comput Biol. 2026 Jan 6;22(1):e1013866. doi: 10.1371/journal.pcbi.1013866 (PMC12799191; doi:10.1371/journal.pcbi.1013866)
Supplement: S2 Algorithm — (PDF) [file pcbi.1013866.s004.pdf]

S2 Algorithm Synthesis of Rat EMG From Binary Motor-Unit Action Potential (MUAP)

Require: Firing matrix $\mathbf{F} \in \{0,1\}^{N\times T}$; sampling rate f_s (Hz); AP→MUAP delay Δ_{ms} (ms)

Ensure: EMG time series $\mathbf{e} \in \mathbb{R}^T$

$\text{dt} \leftarrow 1000/f_s$ ▷ ms per sample
$D \leftarrow \lfloor \Delta_{\text{ms}}/\text{dt} \rfloor$ ▷ sample delay
$\text{logBase} \leftarrow 1.05$
$\overline{L} \leftarrow \lfloor 7.5/\text{dt} \rfloor, \quad \sigma_L \leftarrow \lfloor 2/\text{dt} \rfloor$ ▷ MUAP duration prior (ms)
$N \leftarrow \text{rows}(\mathbf{F}), \quad T \leftarrow \text{cols}(\mathbf{F})$
Sample $L_i \leftarrow \overline{L} + \text{round}(\mathcal{N}(0,\sigma_L))$ for $i = 1..N$
Sample $A_i \leftarrow |1 + \mathcal{N}(0,0.2)|$ for $i = 1..N$
$T_{\max} \leftarrow \max_i L_i$; initialize $\tilde{\mathbf{e}} \in \mathbb{R}^{T+T_{\max}+D}$ with zeros
Construct MUAP shapes
for $i = 1$ **to** N **do**
  $L \leftarrow L_i; \quad n_{40} \leftarrow \lfloor 0.4L \rfloor; \quad n_{60} \leftarrow L - n_{40}$
  $\mathbf{w} \leftarrow (1 - \text{linspace}(0,1,L)^{\circ 2}) \odot [\underbrace{\mathbf{1}}_{n_{40}}, \underbrace{\text{linspace}(1,3,n_{60})^{-1}}_{n_{60}}]$ ▷ envelope
  $\boldsymbol{\phi} \leftarrow \dfrac{\log(\text{linspace}(1, \text{logBase}^{4\pi}, L))}{\log(\text{logBase})}$ ▷ phase modulation
  $\mathbf{u}_i \leftarrow A_i \cdot \mathbf{w} \odot \sin(\boldsymbol{\phi})$ ▷ unit MUAP
end for
Place MUAPs at spike times
for $i = 1$ **to** N **do**
  for $t = 1$ **to** T **do**
    if $F_{i,t} = 1$ **then**
      $\mathcal{S} \leftarrow \{t{+}D, t{+}D{+}1, \ldots, t{+}D{+}L_i{-}1\}$
      $\tilde{\mathbf{e}}[\mathcal{S}] \leftarrow \tilde{\mathbf{e}}[\mathcal{S}] + \mathbf{u}_i$
    end if
  end for
end for
$\mathbf{e} \leftarrow \tilde{\mathbf{e}}[1{:}T]$ ▷ truncate to original length
return $\mathbf{e}$

Notes. (i) MUAP duration L_i is sampled about 7.5 ms with ±2 ms variability (127); (ii) $\mathbf{w}$ yields a biphasic, tapered MUAP envelope, while $\boldsymbol{\phi}$ enforces a smoothly increasing instantaneous frequency using $\text{logBase} = 1.05$; (iii) Complexity is $O(\sum_i K_i L_i)$ where K_i is the number of spikes for unit i (event-driven). Pipeline follows (74).
