# Supplementary material for: Biologically-constrained spiking neural network for neuromodulation in locomotor recovery after spinal cord injury
Source: PLoS Comput Biol. 2026 Jan 6;22(1):e1013866. doi: 10.1371/journal.pcbi.1013866 (PMC12799191; doi:10.1371/journal.pcbi.1013866)
Supplement: S2 Table — (PDF) [file pcbi.1013866.s006.pdf]

S2 Table. Bayesian Linear Mixture Model checks for convergence across all conditions. Each value must reach the criteria to be considered converged and indicate goodness of fit. Bayesian fraction of missing information (BFMI) quantifies how well momentum sampling matches the marginal energy distribution where close matching distributions are close to 1. Divergences quantify number of discarded samples, resulting in fewer samples for representation. The Gelman-Rubin statistic ($\hat{R}$) assesses convergence within multiple Markov Chain Monte Carlo chains, values close to 1 indicate convergence.

	Value
BFMI_{min}	0.91
Divergences	0.00
$\hat{R}$	1.01
