# Supplementary material for: Biologically-constrained spiking neural network for neuromodulation in locomotor recovery after spinal cord injury
Source: PLoS Comput Biol. 2026 Jan 6;22(1):e1013866. doi: 10.1371/journal.pcbi.1013866 (PMC12799191; doi:10.1371/journal.pcbi.1013866)
Supplement: S3 Table — Equivalence was established across seeds if P(|σ|<ϵ)>0.9. (PDF) [file pcbi.1013866.s007.pdf]

S3 Table. A hierarchical Linear Mixed Model (LMM) was created for whole step extracted firing rate. The x-axis and y-axis were set to steps across time and mean firing rates respectively for each seed. The LMMs were fitted for each condition over all seeds. The max value, standard deviation of the model's errors (σ), and the p-value for equivalence for the slope and intercept were calculated. σ_{dyn} represents the standard deviation of the seed-level dynamic deviations in each condition. This measures how much the dynamic trajectories across steps for different steps differ from one another within the same condition. σ_{int} represents the standard deviation of the seed-level intercept deviations within each condition. Equivalence was established across seeds if P$(|\sigma| < \epsilon) > 0.9$.

Simulated Condition	Max	σ_{dyn}	p_{dyn}	σ_{int}	p_{int}
Baseline	101.72	2.91	0.99	2.90	0.97
SCI	6.85	0.36	0.93	0.43	1.00
$SCI_{5\text{-}HT}$	17.96	0.38	0.92	0.43	1.00
$SCI_{5\text{-}HT+ES}$	0.36	0.37	0.93	0.43	1.00
BWS_{ES}	114.71	3.06	1.00	1.21	1.00
$BWS_{5\text{-}HT}$	317.47	12.89	1.00	0.87	1.00
$BWS_{5\text{-}HT+ES}$	149.82	4.75	1.00	0.87	1.00
