# Supplementary material for: Biologically-constrained spiking neural network for neuromodulation in locomotor recovery after spinal cord injury
Source: PLoS Comput Biol. 2026 Jan 6;22(1):e1013866. doi: 10.1371/journal.pcbi.1013866 (PMC12799191; doi:10.1371/journal.pcbi.1013866)
Supplement: S4 Table — (PDF) [file pcbi.1013866.s010.pdf]

S4 Table. Results of post-hoc pairwise comparisons between simulated conditions using seed aggregated step firing rate samples in the TA MN during stance and swing phase and their difference. Statistical significance and equivalence were assessed for each pair using (1) the two one-sided test (TOST) procedure on mean firing rates to evaluate statistical equivalence ($\epsilon = 15$Hz), (2) a paired t-test (t). Pairs identified as statistically equivalent based on the TOST procedure are indicated in **bold**. All tests were conducted at an $\alpha = 0.05$ significance level.

Phase	Condition A	Condition B	p_{TOST}	p_t
Stance	Baseline	SCI	1.000	0.000
	Baseline	$SCI_{5\text{-}HT}$	1.000	0.000
	Baseline	BWS_{ES}	**0.008**	0.000
	Baseline	$BWS_{5\text{-}HT}$	1.000	0.000
	Baseline	$BWS_{5\text{-}HT+ES}$	1.000	0.000
	SCI	$SCI_{5\text{-}HT}$	**0.000**	0.000
	SCI	BWS_{ES}	1.000	0.000
	SCI	$BWS_{5\text{-}HT}$	1.000	0.000
	SCI	$BWS_{5\text{-}HT+ES}$	1.000	0.000
	$SCI_{5\text{-}HT}$	BWS_{ES}	1.000	0.000
	$SCI_{5\text{-}HT}$	$BWS_{5\text{-}HT}$	1.000	0.000
	$SCI_{5\text{-}HT}$	$BWS_{5\text{-}HT+ES}$	1.000	0.000
	BWS_{ES}	$BWS_{5\text{-}HT}$	1.000	0.000
	BWS_{ES}	$BWS_{5\text{-}HT+ES}$	1.000	0.000
	$BWS_{5\text{-}HT}$	$BWS_{5\text{-}HT+ES}$	1.000	0.000
Swing	Baseline	SCI	1.000	0.000
	Baseline	$SCI_{5\text{-}HT}$	1.000	0.000
	Baseline	BWS_{ES}	**0.000**	0.561
	Baseline	$BWS_{5\text{-}HT}$	1.000	0.000
	Baseline	$BWS_{5\text{-}HT+ES}$	1.000	0.000
	SCI	$SCI_{5\text{-}HT}$	**0.000**	0.000
	SCI	BWS_{ES}	1.000	0.000
	SCI	$BWS_{5\text{-}HT}$	1.000	0.000
	SCI	$BWS_{5\text{-}HT+ES}$	1.000	0.000
	$SCI_{5\text{-}HT}$	BWS_{ES}	1.000	0.000
	$SCI_{5\text{-}HT}$	$BWS_{5\text{-}HT}$	1.000	0.000
	$SCI_{5\text{-}HT}$	$BWS_{5\text{-}HT+ES}$	1.000	0.000
	BWS_{ES}	$BWS_{5\text{-}HT}$	1.000	0.000
	BWS_{ES}	$BWS_{5\text{-}HT+ES}$	1.000	0.000
	$BWS_{5\text{-}HT}$	$BWS_{5\text{-}HT+ES}$	1.000	0.000
ΔPhase	Baseline	SCI	1.000	0.000
	Baseline	$SCI_{5\text{-}HT}$	1.000	0.000
	Baseline	BWS_{ES}	0.303	0.000
	Baseline	$BWS_{5\text{-}HT}$	0.986	0.000
	Baseline	$BWS_{5\text{-}HT+ES}$	**0.001**	0.004
	SCI	$SCI_{5\text{-}HT}$	**0.000**	0.000
	SCI	BWS_{ES}	0.636	0.000
	SCI	$BWS_{5\text{-}HT}$	1.000	0.000
	SCI	$BWS_{5\text{-}HT+ES}$	0.998	0.000
	$SCI_{5\text{-}HT}$	BWS_{ES}	0.265	0.000
	$SCI_{5\text{-}HT}$	$BWS_{5\text{-}HT}$	1.000	0.000
	$SCI_{5\text{-}HT}$	$BWS_{5\text{-}HT+ES}$	0.985	0.000
	BWS_{ES}	$BWS_{5\text{-}HT}$	1.000	0.000
	BWS_{ES}	$BWS_{5\text{-}HT+ES}$	**0.000**	0.000
	$BWS_{5\text{-}HT}$	$BWS_{5\text{-}HT+ES}$	1.000	0.000
