# Supplementary material for: Biologically-constrained spiking neural network for neuromodulation in locomotor recovery after spinal cord injury
Source: PLoS Comput Biol. 2026 Jan 6;22(1):e1013866. doi: 10.1371/journal.pcbi.1013866 (PMC12799191; doi:10.1371/journal.pcbi.1013866)
Supplement: S5 Table — Reported values include the mean, standard deviation (SD), and kurtosis for each locomotor phase (stance and swing) under all experimental conditions. (PDF) [file pcbi.1013866.s012.pdf]

S5 Table. Descriptive statistics of phase-specific firing rates aggregated over seeds across experimental conditions for V2a and GABA interneurons (INs) following z-score based outlier removal (values exceeding ± 3 standard deviations, were removed). Reported values include the mean, standard deviation (SD), and kurtosis for each locomotor phase (stance and swing) under all experimental conditions.

Neuron Type	Phase	Simulation Condition	Mean	SD	Kurtosis
V2a IN	Stance	Baseline	26.10	4.58	-0.22
		SCI	26.10	4.58	-0.22
		$SCI_{5\text{-}HT}$	38.16	4.52	-0.20
		$SCI_{5\text{-}HT+ES}$	56.49	4.14	-0.07
		BWS_{ES}	42.86	4.41	-0.06
		$BWS_{5\text{-}HT}$	30.76	4.58	-0.20
		$BWS_{5\text{-}HT+ES}$	49.93	4.25	-0.04
	Swing	Baseline	28.33	3.68	-0.18
		SCI	28.33	3.68	-0.18
		$SCI_{5\text{-}HT}$	36.45	3.57	-0.25
		$SCI_{5\text{-}HT+ES}$	49.02	3.62	-0.15
		BWS_{ES}	39.33	3.65	-0.10
		$BWS_{5\text{-}HT}$	31.56	3.57	-0.19
		$BWS_{5\text{-}HT+ES}$	44.30	3.61	-0.15
GABA IN	Stance	Baseline	62.33	4.65	-0.66
		SCI	62.33	4.65	-0.66
		$SCI_{5\text{-}HT}$	62.33	4.65	-0.66
		$SCI_{5\text{-}HT+ES}$	94.31	4.24	-0.67
		BWS_{ES}	38.09	4.56	-0.84
		$BWS_{5\text{-}HT}$	8.75	3.75	-1.02
		$BWS_{5\text{-}HT+ES}$	38.09	4.56	-0.84
	Swing	Baseline	65.28	4.02	-0.09
		SCI	65.28	4.02	-0.09
		$SCI_{5\text{-}HT}$	65.28	4.02	-0.09
		$SCI_{5\text{-}HT+ES}$	96.89	4.01	0.14
		BWS_{ES}	42.35	3.52	-0.28
		$BWS_{5\text{-}HT}$	17.66	2.04	-0.33
		$BWS_{5\text{-}HT+ES}$	42.35	3.52	-0.28
