# Supplementary material for: Biologically-constrained spiking neural network for neuromodulation in locomotor recovery after spinal cord injury
Source: PLoS Comput Biol. 2026 Jan 6;22(1):e1013866. doi: 10.1371/journal.pcbi.1013866 (PMC12799191; doi:10.1371/journal.pcbi.1013866)
Supplement: Attachment — Submitted filename: Review_Neuromodulation_Locomotor_Recovery_SCI.pdf [file pcbi.1013866.s014.pdf]

Review: Biologically-Constrained Spiking Neural Network for Neuromodulation in Locomotor Recovery after Spinal Cord Injury

Article synopsis:
This study implements a computational model of a flexor motor neuron reflex loop using a spiking neural network. It creates a baseline network for both healthy and a network with spinal cord injury (SCI). It then applies three different treatment types to the network to understand the underlying mechanisms - body weight supported (BWS) with electrical stimulation (ES), BWS with a serotonin agonist (5-HT), BWS with 5-HT+ES.

Strengths:
The model is constrained by values found in biological studies and the approach to building the model is based in the literature. The model is able to test different treatment strategies with a direct comparison.

Limitations:
The tests are only run for 9 steps with only one trial per test type.

Major edits:
The study is very interesting and has a clear scientific contribution in relation to understanding SCI treatments. In order to increase clarity about how this model fits in with the current landscape of computational reflex loop models, it would be beneficial to add a short paragraph in the introduction which places this model into the current landscape of similar computational models. For example, comparing to models such as:
Di Russo, A., et al. (2023). Investigating the roles of reflexes and central pattern generators in the control and modulation of human locomotion using a physiologically plausible neuromechanical model. Journal of neural engineering, 20(6), 066006.
This also allows the authors to provide reasoning for the scope of the model within the introduction. As in the above mentioned study, typically the CPG is modeled together with the reflex loop. It is clear that the model is able to provide helpful hypotheses in its current form but the reader would benefit from understanding the reasoning for the choice. The paper does touch on the computational model landscape in the Discussion and that is appreciated, but it would add clarity to provide context within the introduction as well.

The limited value of N for deriving statistics should be addressed. It is ok to retain a 9 step cycle trial period for individual analysis. However, the study would benefit from these 9 step trials being run multiple times. Therefore, statistics can be analyzed within a trial and across trials. This would increase the robustness of the study and provide more evidence for the hypotheses. When only one network setup is used, it is unclear if the model is overfitted. By randomizing setup via initialization from a set of randomly generated seed values, the authors can indicate with more confidence that the model produces an evidence-based hypothesis. As the model is set up with percentage connectivity between populations, this will naturally be altered based on the seed, noise will also change based on the seed value.

Minor edits:
Be sure to define all variables/parameters in text when they are used in tables and equations. For example, Tables 1 and 2 do not have parameters explicitly defined, i.e. I assume that gL is leak conductance but it is not stated.

Figures 3, 11, and 12 should be updated with significance annotations. This way the reader can easily see what is being compared and level of significance is easily understood (i.e with number of stars or ‘ns’ for no significance - *, **, ***, ‘ns’). Furthermore, a table should be included or text added which explicitly states the mean, standard deviation, and p-values from these plots.

When mentioning outliers removal, add the number of standard deviations that was used for this cut off.
