# Supplementary material for: Biologically-constrained spiking neural network for neuromodulation in locomotor recovery after spinal cord injury
Source: PLoS Comput Biol. 2026 Jan 6;22(1):e1013866. doi: 10.1371/journal.pcbi.1013866 (PMC12799191; doi:10.1371/journal.pcbi.1013866)
Supplement: Attachment — Submitted filename: Response_to_Reviewers_PLOS.docx [file pcbi.1013866.s015.docx]

# Response to Reviewers

We thank the Editor and Reviewers for their thorough and constructive comments. We have revised the manuscript to improve clarity, transparency, and reproducibility. The model description has been expanded with detailed justifications, supplementary figures have been appended to the end of the manuscript, and all assumptions are now explicitly explained. Below, we provide a point-by-point response to each comment. Text additions or changes in the revised manuscript are referenced by page number and brief quotations.

## Editor Comments

The reviewers found the topic of your manuscript compelling and recognized potential value of your modeling approach for understanding neuromodulatory interventions after spinal cord injury. However, all three reviewers recommended major revision, citing significant concerns. The most pressing issues include: (1) insufficient model detail and missing figures that currently prevent reproducibility and hinder interpretation; (2) inadequate physiological justification for key modeling assumptions, particularly the representation of neuromodulatory effects and the inclusion of specific neuron types; and (3) overinterpretation of results without sufficient support, especially, but not exclusively, concerning mechanistic claims/conclusions on neuromodulation and release of inhibition.

To move forward, a comprehensive revision is necessary. This should include a full and transparent model description, justification for all assumptions and simplifications, inclusion of all referenced figures, and additional data or simulations to support your conclusions. Please ensure that all reviewer comments, both major and minor, are addressed point by point in your revised submission.

We have fully revised the manuscript to address each concern. The Methods section (p. 3–6) now provides explicit equations and parameters with citations to experimental sources. Physiological justification for neuromodulation effects was expanded on p. 5-6, including the rationale for serotonin-mediated conductance reduction and electrical stimulation modeling. Additional random seed repetitions were added with hierarchical statistical analysis to prove equivalence over random initialisations (p. 6-7 and fig 2). Interpretation in the Discussion (pp. 11–14) was revised to focus on the mechanistic implications supported by simulation results rather than general conclusions. Note that the number of steps has been reduced from 9 to 8. For improved robustness, the first step has been input only as a warm-up for initialisations.

## Reviewer 1

In this computational study, the authors seek to model the impact of presynaptic inhibition of proprioceptive afferent terminals onto motoneurons during locomotor activity in various conditions after spinal cord injury including with epidural stimulation, serotonergist agonism, and body weight support.

The topic is interesting and the model, while simple in its composition, includes physiologically detailed modelling of proprioceptive afferent activity.

We thank the reviewer for their interest in the manuscript and their comments.

**Major issues:**

The inclusion of V2a interneurons is not well motivated in the manuscript

Response: We agree and have expanded the Introduction (p. 2–3) to clarify the functional role of V2a interneurons. We now explain that these neurons mediate left-right coordination, locomotor speeds and are critical for rhythm generation after SCI. The revised text reads: “…identified V2a interneurons (Chx10^+^) as a crucial population for … (p. 2).”

The writing could be improved in the Results section. Results and approaches are often presented without much background or context. For example, why simulate photostimulation of GABA neurons? Those intimately familiar with the study of these neurons may figure it out but other readers will not understand that set of simulations.

We thank the reviewer for their comment and agree further context should be provided. We added rationale before each major analysis. On p. 7, we now introduce the motivation for validating motor evoked potential, photostimulation experiment as a validation of inhibitory control mechanisms, and their respective recruitment protocols. “Afferent fibre and direct motoneuron recruitment curve data from previously validated computational models… Photostimulation paradigms provide an experimentally controlled and reproducible framework…”

The figures can also be improved through the addition of many more simulation results. There are no displays of the simulation output in terms of "locomotor" activity. We are only shown the results of the analysis of that output. It is hard to gauge the physiological relevance of the simulation if we cannot see how the model generates locomotor output

We thank the reviewer for their insight. We have repeated the steps over 32 random seeds and added Figures 2 and 8 to display the TA step activity over time, inter-step variations, and firing rate with EMG overlay.

There is a lot of evidence that spasticity may arise from hyperexcitable motoneurons. To gain a better understanding of how presynaptic inhibition could impact motor activity after spinal cord injury, it would be useful to model possible hyperexcitable motoneurons and see what would be required of presynaptic inhibition to counteract this potential maladaptive motoneuron change.

We thank the reviewer for their insight and agree with their comment. Suggestions to extend the model for hyperexcitable motoneurons and study the presynaptic inhibition required to counteract the spasticity has been added to the future works in p. 14.

What does "target neuron reverse potential" mean? By convention, it is the synapse rather than the neuron that has a reversal potential

We thank the reviewer for spotting this mistake. This has been revised to specify the synapse reversal potential, rather than the neuron reverse potential. It now reads, “The reversal potential of excitatory synapses was set to 0 mV, while inhibitory synapses were set to −75 mV” in p. 5.

Would it be possible to show a f-i curve for the GABA INs and V2a INs to show the consequences of using a AdEx model for those two cell types as opposed the F-i curve of IaINs or MNs? I guess it might be in figure 10, but it is never shown. Also, it is not clear what is the difference between the MN and V2a model. The firing looks tonic for both so not sure why require an AdEx LIF for GABA and V2as?

We thank the reviewer for their suggestion. Figure 1C (p. 3) now includes frequency–current curves for all neuron types, illustrating distinct AdEx vs LIF dynamics.

Figure 2A, can you clarify what is being stimulated in the MEPs? It is all of the sensory afferents?

We thank the reviewer for their question. Additional text has been added to the manuscript to clarify how stimulation was modeled to generate MEPs (p. 7). Recruitment curve data were obtained from a previously published study repository that specified the injected current amplitudes and the number of recruited afferent fibres and motoneurons and provided the data. To reproduce MEPs along a physiologically valid recruitment curve, the same proportional populations were stimulated across corresponding current intensities.

It is not clear in which simulations the GABAergic connectivity to flexor MNs reduced the mean firing rate by 53.6 Hz. Is that in the lower panel of Figure 4? We'd suggest modifying the writing to better walk the reader through the simulations.

We thank the reviewer for their suggestion in improving the reading clarity. Additional tables have been added to clearly indicate the change induced by increased GABA connections in SCI conditions (p 7; table 6).

## Reviewer 2

This study constructs a spiking neural network (SNN) model of the rodent tibialis anterior (TA), incorporating excitatory and inhibitory synaptic interactions, including GABAergic presynaptic inhibition. The neurons are represented by conductance-based, leaky integrate-and-fire (LIF) models. The authors claim to examine the modulatory effects of electrical stimulation (ES) and serotonin agonist (5-HT) in the context of spinal cord injury (SCI), both with and without body-weight-supported (BWS) locomotion, to investigate state-dependent changes in motor neuron excitability. The authors conclude that the synergistic interplay of BWS and ES suppresses excessive stance-induced GABAergic inhibition, shifting the system toward a stable dynamical regime where TA motor neuron firing rates approximate baseline homeostasis. The reviewer is not convinced that single-conductance LIF neuronal models can adequately represent all conditions mentioned, and finds the evidence supporting the article’s claims insufficient. Addressing the concerns outlined in the Major and Minor sections below may change this assessment, but would require a major revision with detailed justification for most of the model’s assumptions.

We thank the reviewer for their time and energy in clearly and critically analysing the manuscript. Several methodological amendments have been made upon reading the reviewers suggestions. We have amended the claims to be more proportional to the evidence and limitations without overreaching. We hope that the changes will be up to standard and address the reviewer’s concerns.

**Major**
The model descriptions lacks sufficient detail, making it impossible to reproduce the results based on the provided description. Additionally, understanding the findings is challenging since one of figures (figure 9) is missing, which appears essential for understanding the results. Providing more detailed methodology and including these figures would greatly enhance clarity and help reproducibility

We thank the reviewer for their insight. The Methods section (pp. 4–6) now provides full mathematical equations, parameter tables (Tables 2–5), and explicit simulation settings. All code is publicly available (GitHub link, p. 4). Finally, previously excluded supplementary figures have been appended to the end of the manuscript and the referencing now specifically uses S# Figure/Table notation.

The claim that the “biologically constrained spiking neural network (SNN) was developed” should be supported by the list of the parameters/constrains taken from the experimental measurements. The term 'biologically constrained' appears nine times in the manuscript, each followed by references. However, the manuscript lacks detailed descriptions of the specific parameters adopted or modifications made based on the referenced experimental data. I would also like to emphasize the importance of clearly distinguishing in the text between measured values and assumed or tuned model parameters from those references.

We thank the reviewer for their attention and feedback. We agree that the parameters between tuning and those taken from experiments should be clearly distinguished. Tables 1 – 5 now clarify the definition of the variables and the reference that was used to set the respective values (p. 4–6).

Formal Definition of “a baseline level of locomotive activity” versus an inhibitory-dominant and body-weight supported (BWS) SCI state is needed.

We thank their reviewer for the comment and agree a formal definition was needed. Definitions for the Baseline, SCI, and BWS state has been added to the revised manuscript, “The Baseline state was set by …” (p. 4; Methods).

How is Electrical stimulation modelled? What is the justification?

We thank the reviewer for their question. For MEP validation, suprathreshold stimulation profiles for MEP validation and modelling has been added to page 7, “Afferent fibre and direct motoneuron recruitment curve data from previously validated computational models,…”. For subthreshold stimulation, Poisson distributional firing at 40Hz with 10mV intensity and 0.2ms pulse widths across all afferent fibres was used (page 5). Previous experiments demonstrate asynchronous exogenous excitatory inputs in cortical structures (Softky and Koch, 1993; Wang et al., 2022).

“Ia and II TA and gastrocnemius medialis (GM) muscle afferent signals were calculated by using musculoskeletal and muscle spindle models during locomotion, described in previously validated computational models (62).” The details must be provided

We thank the reviewer for their comment and agree clarity must be provided. Signals were retrieved from the publicly accessible GitHub repository as per original publication (Formento et al., 2018), this was made explicit in p. 4 and p. 7.

“The present study describes, for the first time, the release of GABAergic inhibition on flexor MNs as a potential mechanism underlying BWS treadmill training.” This claim must be clearly supported by the results. This reviewer failed to find the evidence.

We thank the reviewer for their comment and agree the claim was not clearly supported. The interpretation of the results has since been rephrased to more closely match the outputs (page 1).

“The results demonstrate the synaptic mechanisms by which neuromodulatory therapy tunes the excitation and inhibition of ankle flexor MNs during locomotion for smoother and more coordinated movement.” The modeling data should be described in sufficient detail for a reader to agree with this conclusion

We thank the reviewer for their comment and agree the claim was not supported by sufficient detail. The sentence has since been re-worded to be closer to the reported results (p. 1).

In the model descriptions the equations do not have subscriptions referring to a specific neuron in the circuit and this is impossible to follow. What is the meaning of the variable u in (5) and w in (6)? Is this the same variable?

We thank the reviewer for their comments and questions. The variables have been more clearly defined (Table 1; p. 4) and differentiated across equations. Subscripts where relevant have been added for clarity (p. 4 – 6).

Is capital Vth in tables correspond to low case v^th in equation 5? Where is Vrest and τrefractory used? Could full description of a LIF model be provided for each neuron type so the models could be reproduced? What is the meaning of the variable a?

We thank the reviewer for their comments and questions. We have added a definition table (Table 1; p. 4) and subscripts for clarity and differentiated across equations (p. 4 – 6). A full algorithmic description of a LIF model has been added to the supplemental information section (algorithm 1; p. 19). We hope that this assists in clarity.

“See Fig. 9 for MN responses with varying stimulation pulse widths.” Figure 9 is missing.
Page 7: “Refer to Fig. 1 A for an illustration of the entire network and figures 9 and 10 for tonic bursting neural firing.” The figures 9 and 10 are missing

We thank the reviewers for the comment and apologise for the confusion. The supplemental information has been appended to the end of the manuscript, and the naming convention matches the PLOS Computational Biology standard. Please refer to p. 21 - 22.

Page 7. “To simulate serotonin agonist response, MN gL was reduced by 40% during SCI experiments and 15% during BWS experiments.” The justification of these assumptions is needed

We thank the reviewer for their comment and agree that justification from the references was needed. We have added a paragraph to justify the single parameter surrogate for net increased excitability in p. 5. “To capture the net excitability increase …”.

Page 7. “See Fig. 9 for MN responses with varying stimulation pulse widths.” The figure 9 is missing.

We thank the reviewers for the comment and apologise for the confusion. The supplemental information has been appended to the end of the manuscript, and the naming convention matches the PLOS Computational Biology standard. Please refer to p. 21 - 22.

Equation 7 is not clear. The parentheses which play no role suggest that the equation is not complete.

We thank the reviewer for their comment. This typing error has since been amended (equation 6, p. 5).

Equations describing synaptic currents including the parameters from the table 3 are not provided. Why the table 4 has a parameter values for p? Is this the variable p from the equation 7? Does this contradicts the figure 1 D? In this manuscript, it is a serious problem that the variables representing different entities have the same name.

We thank the reviewer for their comments and questions. We have added a definition table (Table 1; p. 4) and subscripts for clarity and differentiated across equations (p. 4 – 6).

“Synaptic connections, with the exclusion of GABA, were determined by probabilities specified in table 4.” Were these synaptic connections constantly active?

We thank the reviewer for their question. These synaptic connections were set at the beginning of each simulation and kept consistent through the simulated steps. These connections will change according to the condition being tested (with or without SCI). Brian2 can create synapses based on a probabilistic rule. The software can connect all neuron pairs with a probability of p_syn_. This definition has been added in Table 1 and in p. 5, “Synaptic connections between GABA and TA MN indexes…”.

“SCI condition GABA connections were increased by 160%...” Did you increase the conductances?

We thank the reviewer for their question. The conductances for GABA connections were not increased, only the number of synapses were scaled. More detailed description of the process has been added in p.5 for clarification.

GABA connections were tuned to match previous baseline responses (59, 62).” What are these responses? What does it mean? What parameters did you tune and how?

We thank the reviewer for their questions regarding the tuning of GABA connections. The tuned parameters have been more clearly defined in tables 1 – 5. To avoid biasing the model, the population firing rate of MNs were compared against the results of previous computational model outputs (Moraud et al., 2016; Formento et al., 2018). This definition has been included in p. 5, “GABA connections were tuned to qualitatively …”.

“Poisson distributed neural groups simulated subthreshold stimulation with user-specified input rates.” What is a neural group? What is a user-specified input rate? How are is stimulation modeled?

We thank the reviewer for their questions on the model. We hope the following assists with clarification. A neural group is a term used in Brian2 to describe neuron populations. To avoid confusion, this has been reworded to “Poisson distributed stimulus inputs were used to simulate subthreshold ES at a frequency of 40Hz, amplitude of 10mV, and pulse width of…” Further detail on the sub-threshold and suprathreshold stimulation modelling was added (p. 5 and p. 7).

How is EMG signal generated?

We thank the reviewer for their question. A full algorithm (S2 Algorithm) has been detailed in the supplementary information (p. 20).

Figure 2. How was stimulation intensities ranging from 0 – 600 µA encoded in the model?
“Results qualitatively match reported findings.” What do you mean by this?

We thank the reviewer for their question. Additional text has been added to the manuscript to clarify how stimulation was modeled to generate MEPs (p. 7). Recruitment curve data were obtained from a previously published study that specified the injected current amplitudes and the number of recruited afferent fibres and motoneurons. To reproduce MEPs along a physiologically valid recruitment curve, the same proportional populations were stimulated across corresponding current intensities.

Figure 2B. What is depicted on the right panel?

We thank the reviewer for their question. The right panel of Fig 2B depicts the ratio between the control (not stimulated) and photostimulated peaks. Further clarification has been added in the caption to avoid confusion (p. 9).

Page 6. “The ratio of extracellular potential during control and photostimulation conditions was within range of experimental results.” How do you measure the extracellular potential in LIF model?

We thank the reviewer for pointing out this error. This was a typing mistake and has since been amended (p. 8).

“The burst activity during locomotion fell within the range reported in experimental data.” What is the measure used to make this claim?

We thank the reviewer for this question. Greater specificity and direct language between the figure (Fig 3C) and the manuscript body have been provided in p. 8.

Page 6. “Applying 5-HT+ES increased V2a IN and GABA IN activation, reducing the MN excitation to below SCI.” The authors should describe and justify precisely what are the changes in every parameter for each considered condition

We thank the reviewer for their comment and agree. Reference the tables and the results for each condition have now been added to give numerical information. This sentence has been extended to several sentences and reworded for clarity (The facilitatory effects of 5-HT… p.11).

**Minor**
Importance of figure 1C is not clear.

We thank the reviewer for their comment. This figure has now been moved to the supplementary information and replaced with an F-I curve (p. 3).

Velocity in the equation 1 has the same variable name v as the membrane potential in the following equations. Assigning a different letter to it would be helpful

We thank the reviewer for their suggestion. We now use *v*_stretch_ for velocity and V for membrane potential to avoid confusion (Eq. 1, p. 4).

Page 6. “The simulation time step was set to 5 µs and Euler approximations were used for ordinary differential equation solving. A total of 9 steps were simulated, where gait stance and swing phases were split at 65% of the gait cycle (67).” It would be easier to follow if the locomotion steps are distinguished from the time steps in the first sentence.

We thank the reviewer for their suggestion and agree that differentiating the terms would be easier to follow. The text has been updated on p. 2-3.

Page 3.
Cm 0.1 µF/cm2 why is so small? It is 10 time smaller than usually used in models.

We thank the reviewer for pointing out this error. This was a typing mistake and has since been amended (Table 2; p. 5).

Figure 1A The circle marking inhibitory input from GABA to motoneuron collocates with excitatory inputs from Flexor-Ia and V2a creating a confusing marker. Could you make these connections distinguished?

We thank the reviewer for their suggestion and have taken it onboard to distinguish the connections (Fig 1; p. 3).

Figure 1 DE. Could you mark the voltage as a postsynaptic potential?

We thank the reviewer for their suggestion and have renamed the y-axis appropriately (Fig 1; p. 3).

Why -75 mV is chosen as a rest potential? This value does not refer to any neuron in the tables.

We thank the reviewer for pointing out this error. This was a typing mistake and has since been amended (Table 3; p. 6).

Figure 1F. Why time starts from 1 s?

We thank the reviewer for their question. This was a graphical mistake and has since been amended (Fig 1; p. 3).

Figure 1F “Left illustrates the MN extracellular voltage activity with spiking activity indicated with red triangles.” The figure appears illustrating intracellular membrane potential.

We thank the reviewer for pointing out this error. This was a typing mistake and has since been amended (Fig 1; p. 3).

Figure 2C. Remove extra article “the”.

We thank the reviewer for pointing out this error. This was a typing mistake and has since been amended (Fig 3; p. 9).

Page 6. “Simulating SCI serotonergic agonist activity by reducing the membrane conductance of V2a INs and MNs only slightly increased firing rates in MNs.” Why do you simulate “serotonergic agonist activity by reducing the membrane conductance”? Would not agonist activity increase the membrane conductance?

We thank the reviewer for pointing out this error. This was a typing mistake and has since been amended to specify the leak conductance with additional detail on the effect of decreasing leak conductance on excitability (p. 5).

## Reviewer 3

Article synopsis:

This study implements a computational model of a flexor motor neuron reflex loop using a spiking neural network. It creates a baseline network for both healthy and a network with spinal cord injury (SCI). It then applies three different treatment types to the network to understand the underlying mechanisms - body weight supported (BWS) with electrical stimulation (ES), BWS with a serotonin agonist (5-HT), BWS with 5-HT+ES.

Strengths:

The model is constrained by values found in biological studies and the approach to building the model is based in the literature. The model is able to test different treatment strategies with a direct comparison.

Limitations:

The tests are only run for 9 steps with only one trial per test type.

We thank the reviewer for their time and energy in providing constructive feedback and finding interest in the manuscript. We appreciate the recognition of the contribution and addressed each of the reviewer’s comments below.

**Major**

The study is very interesting and has a clear scientific contribution in relation to understanding SCI treatments. In order to increase clarity about how this model fits in with the current landscape of computational reflex loop models, it would be beneficial to add a short paragraph in the introduction which places this model into the current landscape of similar computational models. For example, comparing to models such as:

*Di Russo, A., et al. (2023). Investigating the roles of reflexes and central pattern generators in the control and modulation of human locomotion using a physiologically plausible neuromechanical model. Journal of neural engineering, 20(6), 066006.*

This also allows the authors to provide reasoning for the scope of the model within the introduction. As in the above mentioned study, typically the CPG is modeled together with the reflex loop. It is clear that the model is able to provide helpful hypotheses in its current form but the reader would benefit from understanding the reasoning for the choice. The paper does touch on the computational model landscape in the Discussion and that is appreciated, but it would add clarity to provide context within the introduction as well.

We thank the reviewer for this suggestion and agree with the benefit of adding additional context. We have added a new paragraph to the end of the Introduction that describes how our work fits within the broader context of reflex-loop and CPG-based computational models. Specifically, we reference Di Russo et al. (2023), works by Capogrosso and colleagues, and Danner and colleagues, that integrate CPG and reflex components to highlight the reasoning for our model’s scope. We clarify that the present study focuses on the reflex-mediated modulation of flexor activity under neuromodulatory influences, as a first step toward integrating with more complete CPG frameworks. Please refer to p. 2.

The limited value of N for deriving statistics should be addressed. It is ok to retain a 9 step cycle trial period for individual analysis. However, the study would benefit from these 9 step trials being run multiple times. Therefore, statistics can be analyzed within a trial and across trials. This would increase the robustness of the study and provide more evidence for the hypotheses. When only one network setup is used, it is unclear if the model is overfitted. By randomizing setup via initialization from a set of randomly generated seed values, the authors can indicate with more confidence that the model produces an evidence-based hypothesis. As the model is set up with percentage connectivity between populations, this will naturally be altered based on the seed, noise will also change based on the seed value.

We appreciate this important point regarding statistical robustness and found this comment to be the most helpful insight in improving the manuscript. In the revised manuscript, we added repeated simulations of the model across 32 random seed initialisations, each representing distinct realizations of network connectivity and noise. These seed-level simulations were aggregated using Bayesian hierarchical modeling to assess equivalence and convergence across seeds and steps. This approach ensures that our findings are not dependent on a single trial setup, directly addressing the reviewer’s concern about overfitting. A detailed explanation of statistical inference using Bayesian linear mixed models was provided in p. 6 in the Methods section. The convergence results and the seed equivalence were described in p. 7 in the Results section. Please note that we have reduced the total steps per simulation to eight and have used the first step as a warm-up cycle to set the initial state. Inter-step activity has been added to the results and discussion in Fig 8 and tables 7 – 8.

**Minor edits:**

Be sure to define all variables/parameters in text when they are used in tables and equations. For example, Tables 1 and 2 do not have parameters explicitly defined, i.e. I assume that gL is leak conductance but it is not stated.

We thank the reviewer for their suggestion and have revised all tables and equations to ensure that each variable and parameter is clearly defined in the text or caption. For example, *g_L_* is now explicitly defined as leak conductance, along with all other parameters included in Tables 1.

Figures 3, 11, and 12 should be updated with significance annotations. This way the reader can easily see what is being compared and level of significance is easily understood (i.e with number of stars or ‘ns’ for no significance - *, **, ***, ‘ns’). Furthermore, a table should be included or text added which explicitly states the mean, standard deviation, and p-values from these plots.

We appreciate this helpful recommendation. Figures 5, 8, and Supplementary Figures S5 and S6 have been updated with significance annotations (*, **, ***, and ‘ns’ for non-significant comparisons) for improved clarity. In addition, heatmaps now explicitly report the p-values for all comparisons presented and tables report the mean, standard deviation, and kurtosis (see tables 6 – 8 and supplementary tables 4 – 5).

When mentioning outliers removal, add the number of standard deviations that was used for this cut off.

We thank the reviewer for the suggestion and have clarified the outlier removal procedure in the Methods section. Specifically, we state that data points exceeding three standard deviations from the mean (|z| > 3) were removed prior to statistical analysis (top of p. 7).
