# Supplementary material for: Biologically-constrained spiking neural network for neuromodulation in locomotor recovery after spinal cord injury
Source: PLoS Comput Biol. 2026 Jan 6;22(1):e1013866. doi: 10.1371/journal.pcbi.1013866 (PMC12799191; doi:10.1371/journal.pcbi.1013866)
Supplement: Attachment — Submitted filename: Response to Reviewers.docx [file pcbi.1013866.s016.docx]

# Response to Reviewers

We thank the Editor and Reviewers for their thorough and constructive comments. We have revised the manuscript to improve clarity, transparency, and reproducibility. Below, we provide a point-by-point response to each comment. Text additions or changes in the revised manuscript are referenced by page number.

## Reviewer 1

## The authors have done a lot of work to address the revisions requested. Most of my revisions have been satisfied but there are some issues that remain.

We thank the reviewer for their comments.

Fig. 1C. Not sure what the dots are and the solid lines represent. Are solid lines the fits?

We thank the reviewer for raising this issue. Yes, the solid lines are the fits. The figure subtitle has since been amended to reflect each point as a single data point for the injected current and the solid line as the fits. Please refer to page 3.

Fig. 3B. Not sure what the different lines represent. It makes it hard to compare to the experimental data

We thank the reviewer for highlighting the lack of clarity. The transparent lines indicate a seed specific trial, and the opaque lines indicate the average across seeds. This has been amended on page 9.

The photostimulation is still not motivated well. Is there a citation that can be referenced when this is first introduced in the Methods?

We thank the reviewer for this comment. The description of photostimulation-based validation has been moved to the Methods section and expanded to provide clearer motivation. We now explicitly frame photostimulation as a controlled and reproducible experimental framework, with supporting references to recent work (Gill et al., 2023; Triplett et al., 2025). In addition, we cite a closely related validation approach in which optogenetic perturbations are used to assess the causal validity of biologically constrained spiking network models (Sourmpis et al., 2025). These revisions have been implemented on pages 6 and 7.

"The TA MN population firing rates between Baseline and SCI conditions were significantly different across time
(Fig. 5)". Can you clarify whether you mean Fig. 5A? Makes it easier to see where the info is.

We thank the reviewer for highlighting the lack of clarity in the text. The reviewer is correct in that this line was meant to refer to figure 5A. This has been amended of page 10.

The Results text describing Figure 5 should be improved as it is not clear what figures relate to the conclusions made.

We thank the reviewer for highlighting the lack of clarity in the text. We have added specifications to the sub-figures with respect to Figure 5 to improve clarity on the inferences and conclusions. These changes were made on pages 10 and 11.

# Reviewer 2

Most of my concerns have been addressed, but a few issues remain to be fixed.

We thank the reviewer for their comments.

Concerning the state definitions, the SCI condition is reasonably well-defined with a specific 1.6x increase in GABA IN→TA MN synapses, though the implementation method could be clearer. However, the BWS state lacks a critical quantitative parameter—the actual magnitude of the scalar reduction in Ia and II afferent firing rates—making it insufficiently defined for reproducibility without consulting the cited reference.

We thank the reviewer for highlighting this point. To improve clarity and reproducibility of the BWS condition, we now explicitly state the effective magnitude of the scalar reduction applied to Ia and II afferent firing rates (60% for GM-related afferents and 12.2% for TA-related afferents), corresponding to 60% effective body weight, rather than relying on the cited reference alone. This quantitative definition has been added to the Methods section on page 4.

Table 1: "V Intracellular membrane potential", please, remove intracellular, it is part of the definition of the membrane potential.
Page 5:"AdEx equation was defined per equation (5)." There are two equations marked by (5).

We thank the reviewer for their comment. The equations have now been split and their references corrected. Please refer to page 5.

I think it is more accurate to call periodic spiking during a square pulse of injected current 'tonic spiking' rather than 'tonic bursting.

We thank the reviewer for their comment. We agree with the reviewer and have amended the term to ‘tonic spiking’ on page 5.

Response: "This typing error has since been amended (equation 6, p. 5)." Equation 6 still has parentheses which play no role.

We thank the reviewer for their highlighting this mistake. This equation was missing the min and max clipping function as per the original implementation (Naumann LB and Sprekeler H 2020). This has been added to page 5.

# Reviewer 3

The authors have adequately responded to the initial review and updated the manuscript in a satisfactory manner.

We thank the reviewer for their time and energy in improving the manuscript.
